# Oxidative Stress in the Pathogenesis of Crohn’s Disease and the Interconnection with Immunological Response, Microbiota, External Environmental Factors, and Epigenetics

**DOI:** 10.3390/antiox10010064

**Published:** 2021-01-07

**Authors:** Ester Alemany-Cosme, Esteban Sáez-González, Inés Moret, Beatriz Mateos, Marisa Iborra, Pilar Nos, Juan Sandoval, Belén Beltrán

**Affiliations:** 1 Biomarkers and Precision Medicine Unit, Medical Research Institute Hospital La Fe (IIS La Fe), 46026 Valencia, Spain; ester_alemany@iislafe.es; 2 Inflammatory Bowel Disease Research Group, Medical Research Institute Hospital La Fe (IIS La Fe), 46026 Valencia, Spain; saez_estgon@gva.es (E.S.-G.); ines.moret@uv.es (I.M.); beatriz_mateos@externos.iislafe.es (B.M.); iborra_mis@gva.es (M.I.); nos_pil@gva.es (P.N.); 3 Center for Biomedical Research and Network in the Thematic Area of Liver and Digestive Diseases (CIBEREHD), 28029 Madrid, Spain; 4 Epigenomics Core Facility, Medical Research Institute Hospital La Fe (IIS La Fe), 46026 Valencia, Spain

**Keywords:** Crohn’s disease, oxidative stress, antioxidants, pathogenesis, inflammation, microbiota, dysbiosis, environmental factors, epigenetics

## Abstract

Inflammatory bowel disease (IBD) is a complex multifactorial disorder in which external and environmental factors have a large influence on its onset and development, especially in genetically susceptible individuals. Crohn’s disease (CD), one of the two types of IBD, is characterized by transmural inflammation, which is most frequently located in the region of the terminal ileum. Oxidative stress, caused by an overabundance of reactive oxygen species, is present locally and systemically in patients with CD and appears to be associated with the well-described imbalanced immune response and dysbiosis in the disease. Oxidative stress could also underlie some of the environmental risk factors proposed for CD. Although the exact etiopathology of CD remains unknown, the key role of oxidative stress in the pathogenesis of CD is extensively recognized. Epigenetics can provide a link between environmental factors and genetics, and numerous epigenetic changes associated with certain environmental risk factors, microbiota, and inflammation are reported in CD. Further attention needs to be focused on whether these epigenetic changes also have a primary role in the pathogenesis of CD, along with oxidative stress.

## 1. Introduction

Inflammatory bowel disease (IBD) is a complicated and multifactorial disorder characterized by relapsing and remitting inflammation that can involve the entire gastrointestinal tract. Crohn’s disease (CD) and ulcerative colitis (UC), the two types of IBD, are recognized worldwide as major contributors to gastrointestinal disease. The location of the inflammation and the nature of the histological disorders in the gastrointestinal tract differentiate the two diseases. IBD results from a complex interplay between genetic variation, intestinal microbiota, the host’s immune system, and environmental factors such as drugs, diet, breastfeeding, and smoking, although the exact cause of the disease remains unknown. Environmental/microbiota factors can affect gene expression through epigenetic mechanisms in triggering the disease [1].

The intestinal tract is under continual attack from luminal microbes and from oxidized compounds in the diet, exposing it to recurrent oxidative changes [2]. An imbalance in redox intestinal homeostasis impairs the intestinal epithelial cells and the permeable barrier, activating dysfunctional immune responses [3,4]. Intestinal cells are the key elements in regulating the traffic of antigens toward gut-associated lymphoid tissues; discriminating between commensal and pathogenic antigens; and acting as a crossroad between immunological tolerance and the immune response. Immunological tolerance describes a diverse range of host processes that prevent potentially harmful immune responses within that host. The loss of immune tolerance allows for an exaggerated and harmful immune response [3,4,5]. Cell inflammation and oxidative reactions with the overproduction of reactive oxygen species (ROS) through activated leukocytes can overwhelm the tissue’s antioxidant defenses and can contribute to the functional impairment of the enteric mucosa. This leads to an aberrant response to the luminal agents and the development of chronic abnormal inflammatory and dysfunctional immune responses [6]. An antioxidant intestinal environment reflects the intestinal mucosa’s response, aimed at preventing oxidative damage, and is maintained by a complex dynamic recycling system in which different molecules undergo well-established oxidation–reduction reactions. A proper dietary intake of antioxidants is therefore essential for maintaining low intracellular levels of oxidative species, thereby maintaining a proper gastrointestinal redox balance [2]. Dietary compounds are therefore an important aspect of intestinal health. 

Oxidative stress is reported as a pivotal factor in the pathogenesis of IBD and might be a key effector mechanism leading to cellular/molecular damage and tissue injury. There is evidence that ROS are involved in intracellular signaling and in the regulation of growth, differentiation, and cell death, as well as in inflammation [3,4]. Cells’ antioxidant defenses (mainly molecules and antioxidant enzymes) avoid accumulation and the consequent cell damage is promoted by ROS. Oxidative damage was detected not only in the intestinal mucosa of patients with CD but also in peripheral blood leukocytes [5]. The immune cells that reach the mucosa in CD release a number of ROS that are potentially detrimental. The main pathological feature of CD is an infiltration of polymorphonuclear neutrophils and mononuclear cells into the affected intestinal tract. Neutrophils and other leukocytes produce noxious substances, including ROS and proinflammatory cytokines, such as interleukin (IL)-1, IL-8, and tumor necrosis factor alpha (TNF-α). An imbalance in proinflammatory and anti-inflammatory cytokine levels was shown to occur in CD [2,6]. Similarly, plasma antioxidant defenses are diminished in CD [7].

The role of oxidative stress as a potential etiological or triggering factor for IBD is the subject of increasing interest in recent years. Our group previously characterized the ROS implicated in the oxidative damage occurring in the peripheral blood of patients with CD at the beginning of the disease, prior to any treatment, as well as their antioxidative stress status and possible implications in regulating the processes in CD [6]. Mitochondria are the main organelles responsible for ROS production during physiological and pathological states. Mitochondrial dysfunction could therefore involve a combination of excess ROS production and diminished antioxidant capacity. Oxidative stress leads to mucosal layer damage and bacterial invasion, which in turn further stimulate the immune response and contribute to disease progression [7]. Environmental factors and oxidative stress can affect the disease through epigenetics. Increasing evidence suggests that oxidative stress globally affects the chromatin structure and the enzymatic and nonenzymatic post-translational modification of histones and DNA-binding proteins. A better understanding of diet–host–microbiota–environmental interactions is essential for unraveling the complex molecular basis of epigenetic and genetic interactions underlying the pathogenesis of IBD, as well as the role of oxidative stress in this complex disease.

The aim of this review is to summarize the main findings regarding the oxidant and antioxidant mechanisms involved in CD, their role in the immunological response, the environment’s effects on oxidative stress status, and its involvement in epigenetic changes/modifications.

## 2. Oxidative Stress in Crohn’s Disease

### 2.1. Oxidative Stress and the Impaired Immunological Response

**Figure 1:**
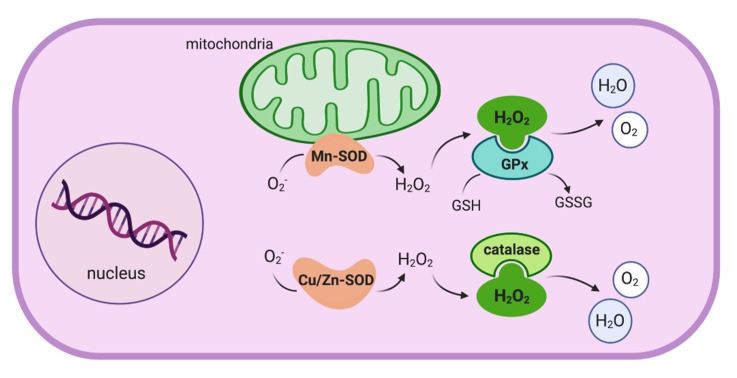
Intracellular antioxidant enzymes responsible for the detoxification of mitochondrial-generated reactive oxygen species. Note that there are two forms of intracellular superoxide dismutase in humans—mitochondrial (Mn-SOD) and cytosolic (Cu/Zn-SOD). These enzymes catalyze the dismutation of the highly reactive superoxide anion (O_2_^−^) to oxygen and hydrogen peroxide (H_2_O_2_). In turn, H_2_O_2_ serves as a substrate for both glutathione peroxidase (GPx) and catalase, which catalyze its reduction to water (figure modified from Moret I [5]).

Oxidative stress, defined as the state in which the oxidant–antioxidant homeostasis within the cell is disturbed, results from an imbalance between ROS production and the defensive system responsible for its detoxification in cells. ROS are natural by-products that include both radical and non-radical oxygen-containing molecules and are mainly produced by mitochondria during oxygen metabolism and water generation. ROS have several physiological roles (e.g., cell signaling regulating growth, differentiation, apoptosis, and inflammatory processes) [8]. However, an increase in the number of these highly reactive molecules in the state of oxidative stress can cause damage to cell components, especially membrane lipids, DNA, and proteins. The main pro-oxidant species are the ROS formed by unstable forms of oxygen—superoxide anion, hydrogen peroxide (H_2_O_2_), and hydroxyl radicals. In contrast, antioxidant agents include both enzymatic and nonenzymatic elements. Antioxidant enzymes are present in all cells, have a primary role in detoxification, and include the enzymes catalase, superoxide dismutase (SOD), and glutathione peroxidase (GPx) (Figure 1). Nonenzymatic antioxidants are usually located in extracellular compartments and include various molecules, such as glutathione, ascorbic acid, and vitamin E [3,9]. 

Inflammation, the main pathological characteristic of IBD, is a process strongly linked to the generation of reactive metabolites, such as reactive nitrogen species (RNS) and ROS [10]. In CD, a massive infiltration of inflammatory cells (polymorphonuclear neutrophils and mononuclear cells) into the affected gut mucosa is reported. The activated neutrophils and macrophages that reach the mucosa stimulate the production of reactive species, including ROS, which are potentially detrimental because they can lead to oxidative stress, causing further inflammation and tissue injury [11,12,13]. In terms of adaptive immunity in CD, the intestinal mucosa accumulates CD4+ T cells in the lamina propria, with an immunological response of the Th1/Th17 type. These cells are resistant to apoptosis, which perpetuates the inflammatory response in the intestinal epithelium. In CD, there is an increased mucosal concentration of the proinflammatory cytokine TNF-α (even during disease remission) [14]. A study reported that numerous apoptotic stimulators, similar to TNF-α, can induce ROS generation by interacting with the respiratory chain in the mitochondria and that these ROS could be acting as mediators in apoptotic pathways [4]. Antioxidant production is the first-line defense against oxidative agents in cells; however, persistent oxidative stress can delete antioxidant cell resources and the ability to produce more antioxidants [15]. In fact, studies as far back as 2003 proposed an imbalanced and inefficient endogenous antioxidant response in the intestinal mucosa of patients with IBD [16,17]. Patients with CD show reduced activity in the main cellular antioxidant enzymes SOD and GPx, as well as reduced levels of the plasma antioxidants vitamin A, C, E, and beta-carotene in the blood and mucosa [15]. Although it is important to note that there is conflicting evidence regarding the change in antioxidant levels, the key point is that there is an imbalance in antioxidant concentrations. It is generally believed that there is excessive oxidant activity and a lower response by antioxidative compounds in CD, which sustain the oxidative stress in the disease [11,18,19].

Considerable evidence strongly suggests that the oxidative stress is coupled with an impaired inflammatory response and chronic inflammation in CD. At the molecular level, oxidative stress and redox signaling are closely involved in the upregulation of inflammatory cytokines and the increased infiltration of inflammatory cells, via the stimulation of signaling pathways (especially the redox-sensitive transcription factor, nuclear factor kappa B). Moreover, inflammation increases oxidative stress by stimulating the ROS/RNS generating systems, along with the release of myeloperoxidase from inflammatory cells [10].

At the clinical level, a recent study [20] found a positive correlation between the oxidative stress index (a general indicator of oxidative stress) and the C-reactive protein levels (a marker of inflammation) in patients with CD, indicating a putative association between higher oxidative stress levels and increased inflammation. As the authors stated, this association could be supported by a previous study [21] in which the redox status of glutathione was heavily reduced (due to increased oxidized glutathione levels in areas of inflammation, indicating greater oxidative stress) in the inflamed ileum mucosa, compared to the noninflamed tissue of patients with CD. Similarly, in a study by Iantomasi et al. [22], higher levels of oxidized glutathione were detected in the diseased ileum than in the healthy ileum of patients with CD. However, this study and the one by Kruidenier et al. [23] reported an increase in the GPx activity (indicating antioxidant capacity) in the inflamed intestinal CD mucosa compared to the controls. A more recent study [24], however, showed reduced GPx activity in the inflamed mucosa compared to either the noninflamed CD mucosa or the healthy controls. The study also mentioned the possible methodological limitations of the previously mentioned studies. Our group established another clinical link between oxidative stress and inflammation. We found an increase in H_2_O_2_ in peripheral lymphocytes and monocytes that correlates significantly with certain inflammation markers (such as C-reactive protein and fibrinogen) during active CD, indicating that the inflammation is more pronounced as the H_2_O_2_ concentration increases in these cells. We also showed that the mitochondrial membrane potential is significantly inhibited in the immune cells (which suggests a mitochondrial source of ROS) and correlates negatively with inflammation markers [6]. The latest clinical evidence of the connection between oxidative stress and inflammation comes from Bourgonje et al. [25], who reported that plasma-free thiols (which reflect systemic oxidative stress, given that they are prime substrates for ROS) showed a negative correlation with inflammation biomarkers and were associated with favorable outcomes in CD.

Subclinical intestinal inflammation is present in a large proportion of patients with CD, even in clinical remission [26], and a recent report stated that CD in clinical remission is marked by systemic oxidative stress [25]. Although the specific mechanism through which oxidative stress is related to the characteristic inflammation in CD is not completely understood, evidence indicates that oxidative stress could have a significant role in the pathogenesis of CD [19].

### 2.2. Oxidative Stress as a Key Effector Mechanism in CD Pathogenesis 

In the state of oxidative stress, ROS can be harmful to cell components, with especially negative effects on membrane lipids, proteins, and mitochondrial and nuclear DNA. ROS therefore have the potential of contributing to the pathogenesis in CD [3]. Lipid peroxidation caused by ROS alters the normal activity of transmembrane enzymes, membrane transporters, and receptors (by disturbing the hydrophobic lipid–lipid and lipid–protein interaction), consequently disrupting the homeostasis and cell metabolism. The end products of lipid peroxidation can cause protein damage, rendering the proteins useless [27,28]. Pelli et al. showed that excess lipid peroxidation is likely an important pathogenic factor in IBD [29], which was later proposed by Sampietro et al. for CD, in particular [30]. A study also reported that treatment with 4-hydroxynonenal (a lipid peroxidation product) exacerbates colonic inflammation through the activation of toll-like receptor 4 signaling [31]. An upward trend in serum and saliva levels of malondialdehyde (a product of lipid peroxidation) was recently reported (an increase that depends on CD severity), as well as a correlation between malondialdehyde levels and the visible symptoms of inflammation [32]. Oxidative DNA damage can cause various lesions, including single and double-strand breaks, apurinic/apyrimidinic sites, and modified pyrimidines and purines. Although DNA damage can be repaired by cellular mechanisms, chronic exposure to oxidative stress leads to the accumulation of DNA lesions, which can therefore promote mutagenesis, human pathogenesis, and loss of homeostasis [9,33]. Oxidative DNA damage was also proposed as a key player in the pathogenesis of IBD and in the associated carcinogenesis [34]. With regard to protein oxidation, ROS can cause hydroxylation or carbonylation of proteins, which can change their function considerably and even provoke their degradation [35]. Krzystek-Korpacka et al. [36] found that IBD is associated with an enhanced formation of advanced oxidation protein products, which have proinflammatory properties.

The oxidative damage of these macromolecules and the effects of pro-oxidants and antioxidants were studied over the past two decades as potential diagnostic, progression, and prognostic markers in CD, including in a recent systematic review on IBD and CD biomarkers by Krzystek-Korpacka [37]. Although a number of these markers show promise, they are mostly at the early research phase of discovery. However, the large number of studies that related CD to oxidative stress markers is evidence of the predominant role of oxidative stress in the disease.

Another finding that reiterates the primary role of oxidative stress in the pathogenesis of CD is its close connection with the main pathologic features of CD. As stated earlier, oxidative stress is related to inflammation and the immune response in CD. It was suggested that ROS overproduction by peripheral immune cells occurs before the cells reach the intestinal mucosa [6], which would link these ROS with the development of the disease. Moreover, the increased vascular density and pathological tissue hypoxia that also characterize CD might lead to increased ROS production through activation of targets of the hypoxia-inducible factor transcription factor family [38,39]. The inflamed mucosa is therefore continually exposed to the detrimental effects of oxidative substances, eventually leading to extensive cell and tissue damage, which accounts for the disease [25]. 

The development of new therapies targeting oxidative stress in CD also puts into perspective the pathogenic essence of this mechanism underlying the disease. A number of unconventional therapeutic methods with antioxidant effects, such as inhibitors against ROS generation, functional dietary interventions, and substances that activate antioxidant enzymes are under investigation as complementary and alternative treatments for IBD, showing promising results [40], although antioxidant therapy remains controversial [15]. Mainstream IBD treatments focus on reducing inflammation, and mainly consist of immunosuppressants, corticosteroids, and anti-TNF-α antibodies. However, it is noteworthy that the therapeutic effect of these drugs is also due to their antioxidative properties. In fact, immunosuppressants and corticosteroids possess direct free radical-scavenging abilities, and anti-TNF-α antibodies carry an indirect antioxidative effect by reducing TNF-α concentrations [18].

Oxidative stress is associated with diarrhea, a frequent symptom in IBD, given that excessive ROS production might be responsible for the excess electrolyte and water secretion that causes diarrhea [41]. The severe clinical activity in CD is reflected by systemic oxidative stress, which likely contributes to the development of the extraintestinal manifestations commonly observed in CD, such as perianal fistulas and arthritis [42]. Oxidative DNA damage might have a primordial role in the inflammation-associated tumorigenesis observed in certain patients with CD, who are at greater risk of colorectal cancer [34,43], which once again highlights the pathogenic potential of oxidative stress, which could go beyond CD.

## 3. The Role of the Environment in Crohn’s Disease

### 3.1. The “In-Vironment”: The Microbiota

The human gut harbors trillions of microorganisms (including bacteria, viruses, fungi, and protozoa) that constitute the gut microbiota. Intestinal bacteria are the predominant microorganisms in the microbial flora, and more than 99% belong to the Firmicutes, Bacteroidetes, Proteobacteria, or Actinobacteria phylum [44]. The microbiota symbiotically interacts with the host, exerting a variety of beneficial effects that include substrate digestion, nutrient production, metabolism, pathogen protection, and remarkably, the normal structural and functional development of the mucosal immune system [45]. The microbiota influences both the local and the systemic immune responses [46] and has a dynamic composition that changes with age and varies according to environmental factors, which is most evident in diet and food intake patterns [47]. Environmental changes can therefore be reflected through changes in microbiota, which in turn can affect the host’s health, which is why the microbiota can be considered an “in-vironmental” factor—the proximate environmental influence contributes to both health and disease states [48]. 

The important role played by the microbiota in immunological responses is reflected in IBD. Specifically in CD, the microbiota triggers the Th1 response, with the consequent generation of interferon gamma and TNF-α, leading to inflammation and mucosal barrier damage [49]. During mucosal inflammation, intestinal epithelial cells, along with immune cells (mainly macrophages and neutrophils), produce proinflammatory cytokines that induce the production of superoxide anion, nitric oxide, and oxidant peroxynitrite, via the activation of nicotinamide adenine dinucleotide phosphate oxidase and inducible nitric oxide synthase. These reactive species are involved in the initiation and progression of CD [40].

Immune reactivity against microbial-derived antigens is reported in patients with CD. In fact, more than 10 types of antimicrobial serologic antibodies were identified as relevant to CD (such as antibodies against the outer membrane porin C of *Escherichia coli*). These serologic antibodies are associated with a more severe CD phenotype and with a higher risk for surgery [50].

Therefore, an abnormal relationship between the host and microbiota can result in an intestinal immune imbalance in CD. However, it is still unclear whether mucosal tissue damage is the result of an abnormal immune response to a normal microbiota or is the result of a normal immune response against abnormal microbiota (dysbiosis) [51].

Dysbiosis, defined as an unfavorable abnormality in the composition and function of the gut microbiota, disturbs the interaction between the host and microbiota and the host’s immune system. Dysbiosis is associated with several human diseases, including CD, in which it appears to play a pivotal role in the pathogenesis [44]. The intestinal microbiota in CD is therefore characterized by decreased diversity, reduced proportions of Firmicutes, and increased proportions of Proteobacteria and Actinobacteria. Moreover, the microbiota of patients with CD is reported to be overpopulated with bacteria with proinflammatory properties (e.g., *Escherichia* and *Fusobacterium*) and reduced populations of anti-inflammatory bacteria (e.g., *Faecalibacterium*) [52,53]. Dysbiosis causes an alteration in the intercellular tight junctions that maintain the integrity of the intestinal mucosa and its permeability. Consequently, opportunistic pathogens can invade the mucosa, resulting in an activation of mucosal-associated lymphatic tissue and the inflammatory cascade (leukocytes and proinflammatory cytokines), which can cause massive tissue damage [54]. These opportunistic pathogens can therefore provoke ROS overproduction in human mucosal epithelial cells, inducing the overexpression of dual oxidase 2 [55].

The functional composition of the gut microbiome, which can be defined as the set of genomes of the microbiota, can provide a more consistent definition of dysbiosis [56], due to the increased stability that the microbiome exhibits over time and the differences in gut microbiome composition between individuals, in contrast to the similarities in phylogenetic profiling [52]. Metagenomic approaches characterizing the microbiome can provide greater insight into the function of the gut microbiota in disease. Metagenomic studies highlight that microbial metabolic pathways are more consistently perturbed in IBD than organismal abundances [57]. In the cited study, Morgan et al. showed that patients with CD show an increase in glutathione transport gene abundance. Glutathione, produced by Proteobacteria and *Enterococcus*, is involved in the maintenance of bacterial homeostasis during oxidative stress. As the authors noted, an increase in the sulfate transport, cysteine metabolism, and glutathione metabolism observed in the patients with IBD might reflect a mechanism by which the gut microbiome addresses the oxidative stress caused by inflammation.

Microbiota-induced inflammation and oxidative stress caused by ROS overproduction are strongly intertwined in CD, given that they reinforce each other and that both lead to mucosal barrier damage. This, in turn, can lead to increased mucosal permeability and loss of protection, allowing for the invasion of pathogens, which can further stimulate inflammation and ROS production, resulting in a vicious circle. Although it is still unclear whether dysbiosis is a primary or secondary phenomenon in CD, it is believed to have a key role in its pathogenesis [58,59].

Genetic findings in IBD also put the microbiota into the spotlight of disease pathogenesis [56,60]. Genome-wide association studies identified more than 160 genetic loci susceptible to conferring protection from IBD or an increased risk of developing IBD [60]. Most of these genes play an important role in the mucosal barrier function, antimicrobial recognition and function, and immune regulation [61]. Consequently, defects or certain variants of these genes can trigger an abnormal immune response to gut microbiota [56]. In CD, these include nucleotide oligomerization domain 2 (*NOD2*), autophagy-related 16-like 1 (*ATG1GL1*), and immunity-related GTPase M (*IRGM*). *ATG16L1* and *IRGM* are autophagy genes involved in the intracellular processing of bacteria [62,63]. *NOD2* was the first susceptibility gene identified for CD more than a decade ago and is known to stimulate the immune system by acting as an intracellular sensor of bacterial peptidoglycans [64,65]. *NOD2* mutations in patients with CD are associated with diminished mucosal α-defensin expression levels, which are antimicrobial peptides that play an important role in the mucosal antibacterial barrier [66]. In other clinical studies, the presence of *NOD2* risk alleles in patients with IBD was associated with changes in microbial composition, such as an increased number of Actinobacteria and Proteobacteria, leading to the idea that these *NOD2* variants could be contributing to bacterial dysbiosis [67,68]. Many of the genetic loci that confer risk in CD interact with each other, which is the case for *NOD2* and *ATG16L1*. Interestingly, NOD2 activation by bacteria and bacterial ligands provokes the ATG16L1-mediated formation of autophagic vacuoles in both epithelial and dendritic cells [69]. NOD2 thereby controls bacterial infection via the induction of autophagy [70]. It was also recently reported that NOD2 and ATG16L1 might cooperate as part of a common pathway to promote anti-inflammatory immune responses to the microbiota [71]. It is believed that *NOD2* and *ATG16L1* variants associated with CD result in the impaired induction of microbial-stimulated autophagy [51].

Human twin studies have not, however, provided much support for a host genetic influence on the gut microbiota. In fact, healthy siblings of patients with CD show an altered microbial and immune profile associated with CD that differs from their genotype-related risk [72]. Studies on twins also revealed that gastrointestinal microbial populations vary with CD phenotypes [73], which highlights the relevance of the external environment in shaping the microbiota, likely outweighing that of genes. Moreover, external factors strongly linked to changes in the gut microbiota, such as antibiotic therapy, appear to be associated with the development of IBD [74], which supports the idea that changes in the microbiota can act as “translators” of environmental factors in the development and progression of CD.

### 3.2. External and Environmental Factors 

It is well established that external and environmental factors have an important influence on the onset and course of IBD. The fact that approximately two-thirds of patients have no identifiable genetic defects, along with the rapid increase in the incidence and prevalence of the disease (which cannot be due to genomic changes), strengthens this idea [75]. The increasing incidence of IBD in newly industrialized countries and its increasing prevalence in Western countries can be attributed to the influences of a Western lifestyle, urbanization, and industrialization, which were reported as primary risk factors for CD and UC [76]. Studies indicate that the incidence of CD increases in immigrants who migrate from regions with a lower prevalence to regions with a higher prevalence of CD within one or two generations, which further supports the massive influence of the environment on CD pathogenesis [77].

#### 3.2.1. Western Diet Versus the Mediterranean Diet and Their Impact on Crohn’s Disease

Environmental elements, such as diet, can directly affect the epithelial mucosa barrier and immune function and can act indirectly through the modulation of intestinal microbiota [78]. “Westernized diets,” which are rich in saturated fatty acids and n-6 polyunsaturated fatty acids (PUFAs), animal proteins, simple sugars, and refined carbohydrates but have a low fiber content (low vegetable and fruit intake), might be a trigger for CD [79,80]. The quality and quantity of food were shown to affect gut microbiota [48], which could theoretically lead to inflammation in genetically susceptible individuals [51]. 

In a recent study conducted in mice, Agus et al. proved that the Western diet causes an inflammatory environment in the digestive tract associated with microbiome perturbations [81]. Previous studies (also conducted in mice) already indicated that a diet high in fat and sugars induces dysbiosis in the mucosa microbiota and is associated with a less protective mucosal layer and increased permeability, which can result in low-grade inflammation and metabolic disorders [82,83]. As Agus et al. reported, the Western diet could deregulate inflammation in the gut mucosa by affecting short-chain fatty acid (SCFA) production. SCFAs (such as acetate, propionate, and butyrate) are the main end-products of the microbial fermentation of dietary fiber. Butyrate typically constitutes 15%–20% of SCFAs in the human colon, is the predominant energy source for colonocytes, and is thought to promote intestinal barrier protection [84]. Butyrate likely augments the intestinal epithelial barrier function via the stabilization of hypoxia-inducible-factor-1 [85], which regulates the integrity of epithelial tight junctions [86]. Butyrate was also reported to act via activation of AMP-activated protein kinase [87]. Other in vitro studies proposed that low concentrations of butyrate could have a protective effect by increasing the synthesis of mucin 2 (MUC2), the main component of intestinal mucus [88,89]. It was proposed that butyrate could affect *MUC2* transcription via AP-1 and acetylation/methylation of histones at the *MUC2* promoter (a concept that is further discussed in Section 3.3 Epigenetics as a transductor of environmental factors in Crohn’s disease). Patients with metabolic syndrome show increased colonic MUC2 expression, following a diet-induced increase in SCFA and butyrate production [90]. However, in vivo studies on pigs and rodents produced ambiguous results regarding the relationship between luminal butyrate (SCFA) levels and MUC2 abundance [90]. Further research is therefore needed to uncover the butyrate-mediated mechanisms in healthy individuals and in patients with IBD. 

Butyrate is also attributed with anti-inflammatory properties, which could be mediated through the inhibition of nuclear factor-kappa B activation, inhibition of interferon gamma signaling, or the upregulation of peroxisome proliferator-activated receptor gamma [91]. Microbial-derived butyrate also induces functional colonic regulatory T cells [92]. Through metagenomic and proteomic studies, Erickson et al. confirmed the presence of lower overall levels of butyrate and other SCFAs in ileal CD [93]. Geirnaert et al. demonstrated that increased butyrate production by bacteria supplemented in vitro to the microbiota of a patient with CD enhanced intestinal epithelial barrier integrity [94]. A lack of butyrate and other SCFAs could also have indirect negative effects on the intestinal mucosa. By assembling a synthetic gut microbiota from fully sequenced human gut bacteria in gnotobiotic mice, Desai et al. [95] demonstrated that, in the absence of dietary fiber, mucolytic bacteria can use host mucus glycans as a source of energy and become the predominant species within the gut microbiota. Consequently, an abundance of these bacteria causes degradation of the colonic mucus layer and promotes pathogen susceptibility. Thus, an insufficiency in microbial-derived SCFA (especially butyrate) caused by a lack of dietary fiber or by dysbiosis [52] (which can also be due to diet) might be involved in the pathogenesis of CD, given it can result in impaired intestinal barrier function and inflammation.

The Western diet is characterized by a typically high consumption of n-6 PUFAs and a low consumption of chain n-3 PUFAs, leading to an imbalanced n-6/n-3 ratio, with detrimental health consequences [96]. N-6 PUFAs are considered proinflammatory compounds, given that linoleic acid (the major dietary vegetable PUFA) is a precursor for arachidonic acid, which is a precursor of inflammatory mediators such as prostaglandins and leukotrienes. In contrast, n-3 PUFAs appear to be inflammation regulators [97]. The increased consumption of n-6 PUFAs (along with the consumption of animal protein) is related to the increased incidence of CD in Japan [98]. Experimental studies indicated that a nutritional intervention with n-3 PUFAs exerts beneficial effects with regards to intestinal inflammation [97]. However, clinical trials to evaluate the effects of n-3 PUFAs for maintaining remission in CD e found no benefit from free n-3 PUFAs over placebo, on clinical relapse [99,100].

Food additives are another component typically in overabundance in the Western diet that are proposed to be proinflammatory agents. Chassaing et al. [101] conducted in vivo studies with mice and proposed that commonly used emulsifiers can disturb the host–microbiota relationship, resulting in microbiota with increased mucolytic and proinflammatory activity that promote chronic intestinal inflammation, which can manifest as colitis. Another recent study by Mu et al., also conducted in mice [102], concluded that titanium dioxide nanoparticles (another widely used food additive) could interfere with the balance of gut flora and the immune system, cause prolonged low-grade intestinal inflammation, and exacerbate the immunological response.

Unlike the Western diet, the Mediterranean diet is believed to have a positive and anti-inflammatory effect on IBD. The Mediterranean diet is characterized by a high consumption of fruit and vegetables, whole grains, oily fish, olive oil, seeds, and dried fruits [103]. In remarkable contrast to the Western diet, the Mediterranean diet provides fermentable dietary fiber, healthy monounsaturated and polyunsaturated fatty acids, with a balanced n-6/n-3 PUFA ratio, antioxidants, and vitamins originating from minimally or unprocessed food. In a case-control clinical study conducted by Souza et al. [104], a diet pattern based on vegetables, fish, olive oil, fruit, grain, and nuts (i.e., the Mediterranean diet) was inversely associated with CD. Marlow et al. reported that a Mediterranean-inspired diet appeared to benefit the health of patients with CD, showing a trend for reduced inflammation markers and for normalizing the microbiota [105]. A diet rich in vegetables and fibers has a positive impact on the microbiota, given it reduces intestinal pH and prevents the growth of potentially pathogenic bacteria (such as strains of *Escherichia coli* and other *Enterobacteriaceae)* [103]. Mediterranean-style diets also favor the proliferation of beneficial bacteria, such as lactic acid bacteria, through the high consumption of fermented foods and n-3 PUFAs [106]. 

The benefits of this dietary pattern could also be largely due to its antioxidant effects. Extra virgin olive oil, considered the Mediterranean “liquid gold”, is rich in antioxidants (e.g., polyphenols) that cooperate to increase plasma antioxidant capacity. The consumption of this oil increases the antioxidant activity of enzymes such as catalase, SOD, and GPx, which have a primary role in preventing oxidative stress. Studies demonstrated that extra virgin olive oil (in healthy people) modulates the response against oxidative stress through antioxidant enzymes [107]. With even a greater link to IBD, recent studies indicated the strong anti-inflammatory effect of this oil in gut mucosa, due to its synergic action with other antioxidant molecules (such as hydroxytyrosol and squalene) [103]. Fruits and vegetables, also abundant in the Mediterranean diet, are not only a source of fiber (and can therefore favor SCFA production) but are also a source of vitamins, polyphenols, and other antioxidants [108] that can be useful in combating oxidative stress. 

These dietary patterns are therefore an example of how diet can influence the onset and development of CD. Food and nutrients have a huge impact on microbiota, immune response-related pathways, and redox mechanisms. The Western and Mediterranean diets are probably the most studied diets with regard to CD, but despite this, there is a lack of scientific literature and clinical trials addressing their impact on CD. In addition to the Mediterranean diet, studies were conducted on the enteral exclusive nutrition diet, partial enteral nutrition diet, and supplementation with probiotics and antioxidant micronutrients as possible therapeutic strategies against IBD [40,84]. Although the study of the effects of diet is marked by conflicting results, difficulty in establishing solid conclusions, and research that is still to be undertaken, it is clear that diet could be a determinant in the pathogenesis of CD.

#### 3.2.2. Other Lifestyle Factors and Health Conditions Relevant to the Pathogenesis 

A recently published umbrella review of meta-analyses on the environmental risk factors for IBD [109] identified smoking, urban living, and having undergone appendectomy or tonsillectomy as the primary risk factors for CD, whereas physical activity, bed sharing, and high levels of vitamin D reduced the risk. Among these factors, smoking stands out because of its sizeable impact on CD. According to epidemiological data, cigarette smoking is one of the well-established risk factors for CD and probably the most widely investigated environmental factor that influences the course of CD. Smoking is believed to increase susceptibility to CD and aggravate its clinical course [77]. A recent systematic review and meta-analysis [110] revealed that smokers with CD have a more complicated disease course with greater flare-ups of disease activity and higher needs for first and second surgeries. Smokers with active CD were reported to have a clinically relevant dysbiosis of the gut microbiota [111]. Several studies suggested that smoking could suppress the innate immune response to bacteria through the direct inhibition of bacterial sensing patterns such as the recognition of lipopolysaccharide by the TLR4/MD-2 receptor [112]. As Bergeron et al. proposed, the striking anti-inflammatory and immunosuppressive effects observed in patients with CD who smoke, which are associated with compromised regulatory adaptive responses, might render cells more susceptible to persistent inflammatory and oxidant injury. Cells from patients with CD who smoke presented a defective sensitivity to anti-inflammatory or antioxidant protection. Above all, smoking most likely has a major role in promoting oxidative stress in patients with CD. Cigarette smoke affects ROS-generation pathways and has high levels of ROS, peroxynitrite, free radicals, and reactive organic compounds that ultimately produce oxidative stress [113]. The metal ions in tobacco smoke also facilitate the transformation of H_2_O_2_ into highly reactive hydroxyl radicals [40]. Long-term smoke exposure can therefore result in a systemic oxidant–antioxidant imbalance and ultimately systemic oxidative stress [114], which can negatively affect the gastrointestinal tract and promote the development of CD. 

To focus on the impact of cigarette smoking on CD is not only relevant because of its evident negative influence but also because it is a (relatively) easy factor to control/avoid (in comparison to other environmental risk factors such as pollution or stress). All in all, cigarette smoking altogether with the previously discussed dietary patterns are lifestyle habits that could be reverted and which could modulate the predisposition or course of CD (Table 1). 

We extensively discussed the interconnection of the most relevant environmental factors, from the microbiota “in-vironment” to cigarette smoking, with the impaired immunological response, genetic susceptibility, or oxidative stress in CD. However, the limitations that arise from combining such broad topics should be kept in mind, e.g., conflicting results and multiple confounding factors that prevent us from establishing robust conclusions or causative relationships. Moreover, these confounding factors also hinder the design of clinical studies. For example, cigarette smoking or the Western diet might be associated with unhealthier lifestyles; and therefore, the findings obtained when studying their influence on CD could be partly due to other factors (e.g., the amount of exercise practiced or the exposition to sunlight) that can be difficult to take entirely into account in clinical studies. We believe that ongoing research assessing these multivariable factors on CD should try to consider as many confounding aspects as possible, while remaining cautious when establishing conclusions.

### 3.3. Epigenetics as a Transducer of Environmental Factors in Crohn’s Disease 

Epigenetics is an emerging field in biomedicine and refers to the heritable alterations in gene expression that are independent of the DNA sequence. The major epigenetic mechanisms that control gene expression are DNA methylation, histone modifications (such as acetylation and methylation), and small, non-coding RNAs. Epigenetic mechanisms are dynamic, reversible, and influenced by exposure to environmental factors [115]. Given that these mechanisms are involved in proper cell development, differentiation, function, and homeostasis, their dysregulation is proposed to play a key role in the onset and development of several diseases, especially cancer [116]. In terms of IBD, epigenetic mechanisms are shown to play a potentially primary role in its pathogenesis [117,118]. Epigenetics can provide a link between genetics and the environment, including the “in-vironment” (microbiota), acting as a transducer of environmental risk factors or even extending the inflammation and oxidative stress that characterize CD.

Epigenetic mechanisms, especially DNA methylation and microRNA expression, are identified as dysregulated in CD and are proposed as candidate biomarkers of the disease [115]. DNA methylation is the most studied epigenetic modification and consists of the covalent addition of a methyl group to the 5′ carbon of the cytosine ring, in the context of CpG dinucleotides. DNA methylation regulates gene transcription in such a way that the methylation restrains gene expression [119]. Our group recently identified an epigenetic methylation signature that allows for the characterization of patients with CD and supports the involvement of the environment and immune system in the pathogenesis of CD [120]. A previous study defined a global methylation profile characteristic of ileal CD, in which the targets of epigenetic modification appeared to be involved in immunity-related pathways [121]. Blood-derived DNA methylation signatures of CD were described that correlate with the severity of the intestinal inflammation [122]. In the latter study, the DNA methylation signatures were a result of the inflammatory features of the disease (given that, with treatment, the DNA methylation patterns resembled the patterns observed in patients without intestinal inflammation). Moreover, micro-RNAs (miRNAs) are proposed to have a more active role in the pathogenesis of CD. miRNAs are short strands of noncoding RNA that post-transcriptionally regulate gene expression [123]. In the intestinal tract, miRNAs are involved in tissue homeostasis, intestinal cell differentiation, and maintenance of the intestinal barrier function, and they were proposed to be both possible biomarkers and therapeutic targets in IBD [124]. The innate immune response to bacterial infection is regulated by an intricate network of miRNA circuits that fine-tune the inflammatory response. Moreover, miRNAs appear to be involved in the dysregulation of autophagy and Th17 signaling in CD [125].

**Figure 2:**
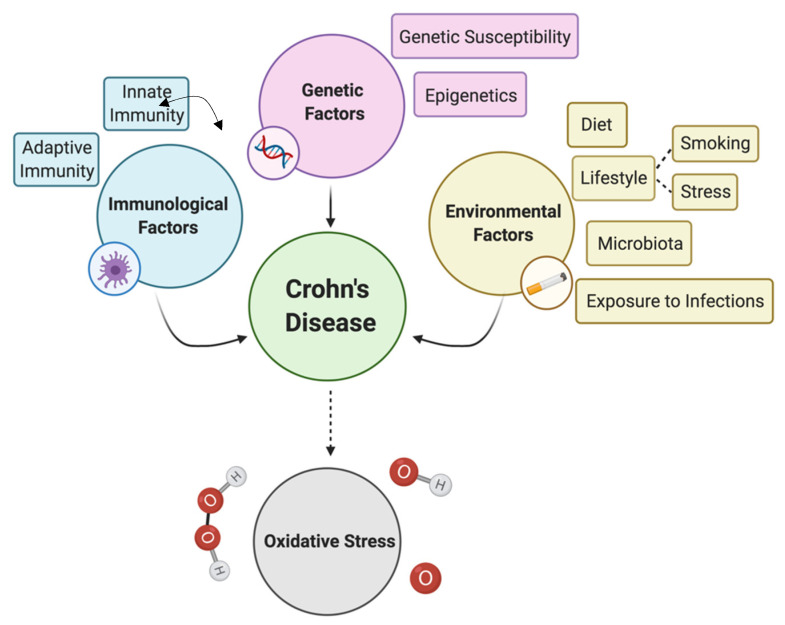
Factors linked to the etiopathogenesis of Crohn’s disease and oxidative stress as a key effector mechanism underlying the pathogenesis. Immunological factors associated with CD comprise both impaired innate response (infiltration of activated neutrophils and macrophages into the affected gut mucosa) and adaptive response (accumulation of CD4^+^ T cells in the lamina propria with a Th1/Th17 immune response). Environmental factors, especially diet, tobacco smoking, and the microbiota (the “in-vironment”), have an outstanding influence on the course and development of CD that appears to outweigh the influence of genetic factors (i.e., genetic susceptibility to the disease). Epigenetics provides a link between genetics and environmental factors and might constitute, at least to some extent, the mechanism through which some environmental factors mediate their impact on CD. Oxidative stress plays a central role in CD pathogenesis and was associated with the aforementioned factors.

Given that environmental factors are known to influence epigenetic regulation, certain environmental risk factors for CD could mediate their negative action, at least to a certain extent, through epigenetics (Figure 2), with an imbalanced diet being one of those risk factors. To cite the most direct example, one-carbon metabolism is dependent on dietary food components (e.g., methionine, betaine, and folate), which participate in DNA methylation pathways and the supply of methyl groups [58]. Considering that the Western diet is often deficient in micronutrients, such as folate, it could provoke a dysregulation of DNA methylation and, consequently, an altered gene transcription profile. Moreover, the low intake of dietary fiber, which can lead to insufficient amounts of microbial-derived butyrate, can also provoke epigenetic dysregulation, which is due to the fact that butyrate is a natural histone deacetylase inhibitor and therefore has the potential to initiate and prolong gene activation. Consequently, butyrate insufficiency could be responsible, to a certain extent, for the excessive condensation of the chromatin structure and gene expression mediated by histone deacetylases [126]. In addition, the putative aforementioned role of butyrate upregulating *MUC2* expression is thought to be partly epigenetically mediated, via the acetylation/methylation of histones at the *MUC2* promoter [88]. In this case, a butyrate insufficiency caused by a poor diet could therefore affect the “normal”/ideal expression of *MUC2* in the intestinal mucosa via epigenetic dysregulation. In vivo studies with rodents showed that the microbiota regulates global histone acetylation and methylation in numerous host tissues in a diet-dependent manner. The consumption of a Western diet prevents many of the microbiota-dependent chromatin changes that occur in a polysaccharide-rich diet [127]. Another well-known environmental risk factor for CD that affects epigenetics is cigarette smoke. Active smoking is an established critical factor for epigenetic modification; alterations in DNA methylation were suggested as a possible mechanism for mediating cigarette smoke-induced diseases [124,125]. 

Environmental factors and oxidative stress can have an impact on disease through epigenetics. Increasing evidence suggests that oxidative stress globally influences the chromatin structure, enzymatic, and nonenzymatic post-translational modifications of histones and the DNA-binding proteins. These chromatin alterations can therefore modulate gene expression, cell death, cell survival, and mutagenesis. Histones are extensively modified in an ROS-dependent and RNS-dependent manner and are glutathionylated in a redox-sensitive manner, which affects their ability to be post-translationally modified [128]. Oxidative stress not only alters global histone modification but also DNA methylation and can therefore have a modulating role in gene expression [129]. Nevertheless, there is a lack of studies addressing whether oxidative stress-induced epigenetic changes can have a further role in the pathogenesis of the diseases characterized by oxidative stress, such as CD, or just constitute collateral changes.

Epigenetic imprinting (not to be confused with genomic imprinting) could be defined as the mechanism through which environmental, external, and “in-vironmental” factors influence epigenetic changes, with potential consequences for health and disease [78]. A great example would be the recently recognized microbiota-sensitive epigenetic signature that predicts inflammation in CD [130]. As the authors indicate, their study defines the manner in which microbiota-derived signals can be integrated by the host via epigenetics, in priming the epithelium for overt clinical disease and with the subsequent disease-associated environmental triggers. Epigenetic imprinting can also act as a “disease memory” and can help explain the relapses, after resections in patients with CD, as well as explain why certain environmental factors appear to influence the intestinal mucosa and disease onset even when the environmental factor is long gone [131]. All in all, it is plausible to believe that the connection between external factors and the host DNA, mediated by epigenetic changes, has a key influence on the phenotypical expression of complex and multifactorial diseases such as CD [58]. However, we need to consider the difficulty in establishing causative relationships and distinguish between epigenetic changes with a possible role in the pathogenesis and those that are merely a consequence of the disease. Future studies should focus on establishing the epigenetic changes that can be derived from environmental risk factors, the microbiota, and even oxidative stress, and those that can contribute to the onset, progression, or relapse of CD. 

## 4. Conclusions

Although the exact etiopathogenesis of CD remains unknown, the role of oxidative stress in its pathogenesis is widely recognized. We discussed how oxidative stress is present in CD, not only locally in the most-affected tissues but also at a systemic level. Oxidative stress is interconnected and feeds back into the impaired immune response and microbiota imbalance in CD.

External and environmental factors are known to have a large influence on the development and course of CD. The aforementioned primary risk factors are also related to oxidative stress, at least to a certain extent. Epigenetics provides a link between genetic and external factors and can provide greater insight into the pathogenesis of the disease. Further studies should seek to determine the environmental and oxidative stress-induced epigenetic changes that could have a role in the onset and development of CD, an area that has much to be explored. 

## Figures and Tables

**Table 1:** Remarkable research conducted in the last decade addressing the impact of lifestyle habits on CD—dietary patterns and cigarette smoking.

Study	Type of Study	Methodology	Main Findings
Agus et al. (2016) [81]	In vivo study	WT and CEABAC10 mice ^1^ were fed with a high-fat/high-sugar diet (HF/HS) (*N* = 6) vs. a conventional diet (*N* = 5) over a period of 18 weeks. Germ-Free (GF) mice were transplanted with fecal pellets of HF/HS donor mice (*N* = 5) or conventional donor mice (*N* = 5), followed by an infection with an adherent-invasive *E. coli* (AEIC) LF82 strain isolated from a CD patient	Western diet causes an inflammatory environment in the digestive tract associated with microbiome perturbations; favors the emergence of *E. coli* associated with the ileal, cecal, and colonic mucosa; and decreases the level of SCFA produced by intestinal microbiota modulating immune response. Transplantation of feces from HF/HS treated mice to GF mice increases susceptibility to AIEC infection
Martinez-Medina, M. et al. (2014) [82]	In vivo study	WT and CEABAC10 mice ^1^ were fed with a HF/HS diet vs. conventional diet for 12 weeks ^2^, and orally infected with AIEC strain LF82	Western diet induces changes in gut microbiota composition with an increase in the mucin-degrading bacterium *Ruminococcus torques* and the group *Bacteroides/Prevotella;* alters intestinal permeability, decreases barrier function, and affects the host homeostasis promoting AEIC gut colonization in genetically susceptible mice
Geirnaert et al. (2017) [94]	Clinical research—In vitro study	Butyrate-producing bacteria supplemented to the fecal microbial communities of CD patients (*N* = 10) in an in vitro system simulating the mucus- and lumen-associated microbiota, and an in vitro study of the resulting microbiota influence on epithelial barrier integrity with a Caco-2 model	In vitro supplementation of microbiota of CD patients with butyrate-producing bacteria results in a higher butyrate production, with and enhanced epithelial barrier integrity in a Caco-2 model. Supports the preclinical development of a probiotic product containing butyrate-producing species
Desai et al. (2016) [95]	In vivo study	Assembled synthetic gut microbiota from fully sequenced human gut bacteria in gnotobiotic mice were fed with fiber-rich vs. fiber-free diets ^2^	In the chronic or intermittent absence of dietary fiber mucolytic bacteria become the predominant species within the gut microbiota with the consequent degradation of the colonic mucus layer and increased pathogen susceptibility
Chassaing et al. (2015) [101]	In vivo study	WT mice and two engineered strains of mice, namely IL10^−/−^ and TLR5^−/−^ (prone to develop shifts in microbiota composition and inflammation) exposed to emulsifiers in the drinking water or to water alone (control group) for 12 weeks ^2^	Relatively low concentrations of commonly used dietary emulsifiers (carboxymethylcellulose and polysorbate-80) can disturb the host-microbiota relationship, induce low-grade inflammation and obesity/metabolic syndrome in WT hosts and promote robust colitis in mice predisposed to this disorder
Mu et al. (2019) [102]	In vivo study	WT mice and DSS-induced colitis mice treated with titanium dioxide nanoparticles vs. standard (control) diet for 3 months from weaning ^2^	First demonstration that long-term dietary intake of titanium dioxide nanoparticles (which are used as food additives) results in lower body weigh along with colorectal inflammation in mice; and it aggravates DSS-induced chronic colitis and immune response *in vivo*, reduces the population of CD4^+^ T cells, regulatory T cells, and macrophages in mesenteric lymph node
Marlow, G. et al. (2013) [105]	Clinical research	6-week intervention with a Mediterranean-inspired diet in CD patients (*N* = 8). Obtention of blood and fecal samples at the beginning and the end of the diet	A Mediterranean-inspired diet appears to benefit the health of CD patients: shows a trend for reducing inflammation and normalizing the microbiota
To, N. et al. (2016) [110]	Systematic review with metanalysis of the effects of smoking on disease course in CD	Search of MEDLINE, EMBASE and EMBASE classic carried out up to July 2015 (with the resulting 33 eligible studies)	Smokers, compared with non-smokers, have 55–85% higher rates of flares of disease activity, clinical recurrence rates after surgery that are two-fold higher, between 54% and 68% higher rates of need for first surgery, and are twice as likely to need a second operation. Quitting smoking appears to have a beneficial effect on CD course, especially for flare of disease activity or need for a second operation
Benjamin, J.L. et al. (2012) [111]	Clinical research	Fecal samples from patients with active CD (*N* = 101; 29 of whom current smokers) and healthy controls (*N* = 66; 8 of whom current smokers) were analyzed by fluorescent in situ hybridization (using probes targeting 16S rRNA of bacteria previously shown to be altered in active CD)	Smokers with active CD have a clinically relevant dysbiosis of the gastrointestinal microbiota; with strong and significant associations between smoking and higher bacteroides (this novel finding is also present in healthy controls)
Bergeron, V. et al. (2012) [112]	Clinical research—In vitro study	Study of mononuclear cells extracted from blood samples of CD patients (smokers *N* = 19, and non-smokers *N* = 26), UC patients (smokers *N* = 7, and non-smokers *N* = 18), and healthy controls (smokers *N* = 13, and non-smokers *N* = 18); following either in vivo or in vitro exposure to cigarette smoke	Mononuclear cells from CD patients who smoke are functionally impaired, present a defective sensitivity to anti-inflammatory or antioxidant protection, and particularly synthesize lower levels of cytoprotective Hsp70. Findings suggest that the effects of cigarette smoke are largely dependent on the oxidative stress generated rather than on the nicotine component

^1^ CEABAC10 mice serve as a model of host susceptibility to adherent-invasive *Escherichia coli* (AIEC) colonization (since they express CEACAM6, which is abnormally expressed in CD patients and predisposes to AIEC colonization). ^2^ In these studies, the N number of mice groups varies among the conducted experiments.
